# Association between Chronic Pain and Diabetes/Prediabetes: A Population-Based Cross-Sectional Survey in Saudi Arabia

**DOI:** 10.1155/2020/8239474

**Published:** 2020-06-24

**Authors:** Khaled K. Aldossari, Mamdouh M. Shubair, Jamaan Al-Zahrani, Abdulrahman A. Alduraywish, Khalid AlAhmary, Salwa Bahkali, Sara M. Aloudah, Sami Almustanyir, Laila Al-Rizqi, Sally A. El-Zahaby, Paivi Toivola, Ashraf El-Metwally

**Affiliations:** ^1^Family& Community Medicine Department, College of Medicine, Prince Sattam Bin Abdulaziz University, Al-Kharj, Saudi Arabia; ^2^School of Health Sciences, University of Northern British Columbia (UNBC), 3333 University Way, Prince George, BC V2N 4Z9, Canada; ^3^Internal Medicine Department, College of Medicine, Jouf University, Sakaka, Saudi Arabia; ^4^College of Public Health and Health Informatics, King Saud Bin Abdulaziz University for Health Sciences, Riyadh, Saudi Arabia; ^5^Princess Nourah Bint Abdulrahman University, King Abdullah Bin AbdulAziz University Hospital, Riyadh, Saudi Arabia; ^6^Department of Family and Community Medicine, College of Medicine, King Saud University, Riyadh 29391, Saudi Arabia; ^7^Ministry of Health, Riyadh, Saudi Arabia; ^8^Internal Medicine, Security Forces Hospital, Riyadh, Saudi Arabia; ^9^Department of Pharmaceutics and Pharmaceutical Technology, Faculty of Pharmacy and Drug Manufacturing, Pharos University in Alexandria, Alexandria, Egypt; ^10^King Abdullah Specialist Children's Hospital, King Abdulaziz Medical City, Riyadh, Saudi Arabia

## Abstract

**Background:**

Diabetes is a debilitating chronic health condition that is associated with certain pain syndromes. The present study sought to evaluate chronic pain and its association with diabetes mellitus at a population level.

**Methods:**

A population-based cross-sectional questionnaire survey study was conducted in Al-Kharj, Saudi Arabia, from January 2016 to June 2016. Participants from both private and governmental institutions were selected following a multistage sampling technique and using a cluster sampling method. Anthropometric measurements were taken, including body weight, height, body mass index (BMI) and waist circumference. A blood sample was also drawn from each respondent for fasting blood sugar, HbA1c, and fasting lipid profile. A *P* value of less than 0.05 indicated statistical significance.

**Results:**

A total of 1003 subjects were included for final analysis. Compared to prediabetic and nondiabetic individuals, diabetic subjects had a higher prevalence of lower limb pain (11.1%), back pain (8.9%), abdominal pain (6.7%), and neck pain (4.4%) (*X*^2^ = 27.792, *P* = 0.015). In a multiple logistic regression model, after adjusting for age, gender, education level, cholesterol, and smoking status, diabetic/prediabetic patients had a significantly higher prevalence of chronic pain ((OR) = 1.931 (95% CI = 1.536–2.362), *P* = 0.037). Increased age was also significantly associated with chronic pain ((OR) = 1.032 (95% CI = 1.010–1.054, *P* = 0.004).

**Conclusion:**

Results of this study found a significant association between diabetes and prediabetes and chronic pain symptoms. Prospective studies are needed to explore temporality of such association.

## 1. Introduction

According to the World Health Organization (WHO), diabetes is defined as a metabolic disorder characterized by high levels of blood glucose that may cause serious damage to many organs, namely, the heart, blood vessels, eyes, kidneys, and nerves. Management of chronic diseases such as diabetes mellitus (DM) not only includes maintaining a normal blood glucose level but also extends to the handling of associated symptoms that can affect different body parts. A substantial amount of money, $174 billion, is reported to be spent in controlling the disease annually [1]. In 2014, about 422 million patients worldwide suffered from diabetes, and this number has increased to 451 million in 2017 [2, 3]. The global prevalence of diabetes among adults (over 18 years of age) has risen from 4.7% in 1980 to 8.5% in 2014 [4].

Diabetes patients often seek healthcare support after experiencing pain, which is clinically a principal subjective symptom [5]. This is also key to discovering prediabetes, also known as intermediate hyperglycemia, and is defined as a blood glucose level that is above normal but below thresholds for diabetes [6]. It is worth mentioning that the conditions of up to 10% of prediabetics develop into diabetes each year [7]. Different kinds of chronic pain are known to afflict millions of patients suffering from diabetes. Young diabetic patients had an increased ten-year cumulative incidence of chronic pain (musculoskeletal) and also higher number of physician consultations as compared to nondiabetic individuals [8]. This undoubtedly necessitates further investigation into the matter. If it is not well-handled, chronic pain may translate into major health problems and therefore an inordinate amount of money will be consumed, adding further strain on the country's economy.

It was reported that 50% of diabetic patients encounter neuropathy. This percentage might be even higher because many patients report painful sensations only after they become unbearable [9]. Neuromusculoskeletal pain in diabetic patients can affect different body parts. When researchers attempt to explain the relationship between diabetes and muscle pain, they always relate this pain to the alteration in the structural matrix and the mechanical properties of periarticular connective tissues, owing to an unusual deposition of collagen [10]. Diabetic patients have altered blood circulation, which might contribute to the degeneration of connective tissue as well [11]. These abnormalities in connective tissues were found to increase the probability of experiencing low back pain and disc prolapse [12]. Moreover, diabetic patients are more likely to experience rheumatic problems such as the low mobility of joints, stiff hand and carpal tunnel syndromes, shoulder capsulitis, and tenosynovitis [13].

While most research studies have discussed neuropathic pain, very little is known about the nonneuropathic pain or chronic pain which may have started in patients prior to the appearance of symptoms of any neuropathic pain [14]. Studies also reported that there is a marked difference between both pains, and it is necessary to identify the specific type of pain for the effective treatment approach [15]. Studies conducted in Finland showed that higher glucose levels in plasma and diabetes both were associated with daily chronic pain in adults [16, 17]. Similarly, another study conducted among the black population of the USA showed significant and independent relationship between pain and HbA1c (>64 mmol/mol), suggesting greater pain with poor HbA1c control [18]. A recent study showed a genetic predisposition between diabetes and lower back pain [19].

Musculoskeletal pain with different locations is more common among female diabetic patients than male patients. This can be attributed to the increased accumulation of glycation end products in both connective tissues and joints. The most popular reported pain locations are the neck, knees and/or hips, shoulders, back, and arms [20]. This usually appears as nonspecific pain of unknown pathology and, nowadays, it represents a major complaint among patients worldwide [21, 22]. Numerous studies were performed on a selected population to relate these musculoskeletal diseases to their relevant risk factors, yet more studies covering many areas must be continued to compare and generalize the situation. Therefore, population-based studies on these disorders are quite necessary. From this need originated the aim of the current study. Previous published studies of this data set gave insight into the prevalence of the diabetes/prediabetes [23] and chronic pain only [24]. However, the present study sought to evaluate chronic pain and its association with diabetes mellitus.

## 2. Methods

### 2.1. Study Design and Sample Selection

This cross-sectional population-based survey study was conducted at Al-Kharj, a Governorate in central Saudi Arabia near the Capital, Al-Riyadh, was selected. It is characterized by how it contains inhabitants with both urban and rural lifestyles. A total of 1,019 diabetics, prediabetics, and nondiabetics responded to the study. This sample population has also been used in some previous published studies [23, 25, 26].

### 2.2. Inclusion and Exclusion Criteria

Diabetic patients represented 4.3% of the total population of 1,019 who responded to the study. The selected age was 18 years old and above. Non-Saudi residents were excluded from the study.

### 2.3. Data Collection

There were six months of data collection, from January 2016 to June 2016 from Al-Kharj. Researchers guided the participants to fill out the questionnaires. Only complete questionnaires were selected for final analysis, and those were found to be 1003.

### 2.4. Sampling Technique

Participants from both private and governmental institutions were selected following a multistage sampling technique and using the cluster sampling method. Clusters of participants from every chosen institution were obtained, and then randomized sampling was performed from these clusters.

### 2.5. Materials and Measures

Data were collected from the participants using a structured questionnaire. Information about sociodemographics (such as age, gender, marital status, and education level) was obtained. Anthropometric measurements such as body weight (kilograms), height (meters), body mass index (BMI), and waist circumference (centimeters) were taken at the time of interview by trained nurses. All the procedures were carried out based on the standards for anthropometric measurements. In addition to this, a blood sample was also drawn from each respondent in order to analyze fasting blood sugar, HbA1c, and fasting lipid profiles. Different pain site responses were collected from the 1,019 participants in Al-Kharj population. The number of males and females who completed the study was 375 and 628, respectively.

### 2.6. Operational Definition

According to the American Diabetic Association 2018, prediabetes was defined as the HbA1c cutoff level of 5.7% to less than 6.5%, while diabetes mellitus was HbA1c that was greater than or equal to 6.5% [27]. Chronic pain was defined as persistent pain that lasted for three months or more [28].

### 2.7. Data Analysis

All data were expressed as mean ± standard deviation, while the chi-square (*X*^2^) test and percentage were used in the case of categorical data. One-way ANOVA was used to detect the significance of the differences. A *P* value of less than 0.05 indicated statistical significance, and statistical analysis was carried out using STATA version 12.1.

### 2.8. Ethical Approval

After explaining about the purpose and procedure of the study, written informed consent was obtained from all participants. The ethical approval for this study was secured from Committee for Scientific Research and Publication, our local institutional review board. The confidentiality of the data was maintained. This study was directed according to the principles established in the Declaration of Helsinki [29].

## 3. Results

The population characteristics of the study, the results related to the prevalence of chronic pain [24], and the prevalence of prediabetes and diabetes mellitus were previously published [23].

### 3.1. Univariate Analysis

The prevalence of diabetic status (categorical variable: diabetic vs prediabetic vs nondiabetic) was compared across all eight (8) pain sites, using a chi-square (*X*^2^) test. The results showed that, compared to prediabetic and nondiabetic individuals, diabetic subjects have a higher prevalence of lower limb pain (11.1%), back pain (8.9%), abdominal pain (6.7%), and neck pain (4.4%). These results were statistically significant as evident by the chi-square test: (*X*^2^) = 27.792, *P*=0.015 (Table 1).

### 3.2. Multivariate Analysis

#### 3.2.1. Multiple Logistic Regression Analyses (Binary Diabetic Class and Body Mass Index/BMI as Predictors for Chronic Pain Binary Variable)

In a new multiple logistic regression model (Table 2), we examined whether diabetic class binary variable (*diabetic/prediabetic* vs *nondiabetic*) and body mass index (BMI) were significantly associated with chronic pain (binary outcome variable: yes/no), after adjusting for age, gender, education level, cholesterol, and smoking status (Table 2). We found that diabetic/prediabetic patients had a significantly higher prevalence of chronic pain ((OR) = 1.931 (95% CI = 1.536–2.362), *P*=0.037). There was also a linear positive significant association between BMI and the prevalence of chronic pain (OR) = 1.025 (95% CI = 1.000–1.050, *P*=0.051). Increased age (in years) was also significantly and positively associated with chronic pain ((OR) = 1.032 (95% CI = 1.010–1.054, *P*=0.004).

## 4. Discussion

This was a cross-sectional observational study, carried out in Al-Kharj, Saudi Arabia. The results of our study showed that chronic pain is associated with diabetes/prediabetes. Chronic pain is a comorbid ailment which can be burdensome in diabetic patients due to symptomatic manifestations along with psychological and physical disability [30, 31]. This is related to the current study and findings of this paper. From the previous evidence, it can be noted that individuals with diabetes are prone to suffering from chronic pain in their lives. A recently published study reported that chronic pain was very common among diabetic patients, which also limited their routine self-care [32]. Two longitudinal studies reported that metabolic diseases like diabetes have a significant association with higher risks of frequent back, neck, and/or shoulder pain [33, 34]. Research has also shown that patients with type-2 diabetes were associated with an increased risk of chronic musculoskeletal complaints [35, 36].

As suggested by our results, more recent evidence has also shown that diabetes and multiple-site pain not only coexist but might even be linked in some ways. It has been postulated that musculoskeletal conditions, especially chronic pain, may increase the risk of developing other chronic diseases [37]. Similarly, as chronic pain puts a substantial risk on health outcomes such as physical activity and dietary habits, these lifestyle changes may in turn induce type-2 diabetes in the patients [38–40]. A recent Nord-Trøndelag health study published in October 2018 revealed that nondiabetic females with chronic lower back pain have a higher risk of developing diabetes in the following 11-year interval [41].

### 4.1. Clinical Significance

Pain is an essential subjective manifestation in diabetes mellitus patients, and it has a substantial influence on the quality of life. The available research literature and present study emphasize the need for understanding the impact of chronic pain in diabetes mellitus. Our study demonstrated the population-based findings and could pave the way for future research in order to develop effective personalized treatment plans.

### 4.2. Limitations

Our study has a few limitations which need to be addressed. First, subjects were required to recall past information to fill the questionnaire; therefore, recall bias could be an issue and could have resulted in underreporting or overreporting. Second, due to the cross-sectional nature of the study (both exposure and outcomes are simultaneously assessed), temporal relationship and causality cannot be ascertained. Third, although we adjusted for confounding variables, we might have missed some residual confounders that could have affected the results. Another limitation of the study is we did not assess the intensity of the pain. Finally, the study included only single geographical location, and similar studies in other geographical areas and other countries might have resulted in different findings. Nevertheless, since chronic diseases such as diabetes mellitus and chronic pain are pandemic phenomena, we strongly believe that our findings are still relevant and appropriately reveal the condition across the globe.

## 5. Conclusion

From the above findings, it can be concluded that prediabetes and diabetes are significantly associated with chronic pain, after adjusting for age, gender, education level, cholesterol, and smoking status. Our study highlights the importance of preventive measures in lessening the prevalence, incidence, and poor outcomes associated with chronic pain among the diabetic population.

## Figures and Tables

**Table 1:** Comparison between diabetic, prediabetics, and nondiabetic subjects according to pain site in the Al-Kharj study (*n* = 1,019).

Pain site	Diabetic (*n* = 45)	Prediabetic (*n* = 231)	Nondiabetic (*n* = 743)	Total (*n* = 1,019)
	*n* (%)	*n* (%)	*n* (%)	*n* (%)
*Pain site*				
No pain	30 (66.7)	183 (79.2)	622 (83.7)	835 (81.9)
Chest pain	0 (0.0)	3 (1.3%)	14 (1.9%)	17 (1.7)
Head pain	0 (0.0)	8 (3.5%)	22 (3.0%)	30 (2.9%)
Back pain	4 (8.9%)	13 (5.6%)	31 (4.2%)	48 (4.7)
Abdominal pain	3 (6.7)	10 (4.3)	27 (3.6)	40 (3.9)
Lower limb pain	5 (11.1)	8 (3.5)	16 (2.2)	29 (2.8)
Upper limb pain	1 (2.2)	1 (0.4)	4 (0.5)	6 (0.6)
Neck pain	2 (4.4)	5 (2.2)	7 (0.9)	14 (1.4)
Total	45 (100)	231 (100)	743 (100)	1019 (100)

**Table 2:** Logistic regression model using BMI and binary diabetic class variable as predictors for chronic pain (binary outcome), after adjusting for sociodemographic and other variables (*n* = 1,031).

Dependent/outcome categorical variable (pain sites)	B	SE of B	Sig.	Exp (B)/odds ratio	95% CI for odds ratio	
					Lower	Upper
Diabetic class (binary)	1.072	0.194	0.037	1.931	1.536	2.362
Body mass index (BMI)	0.024	0.012	0.051	1.025	1.000	1.050
Age	0.031	0.011	0.004	1.032	1.010	1.054
Gender	0.171	0.100	0.400	1.187	0.797	1.767
Education level	0.031	0.100	0.760	1.031	0.847	1.255
Cholesterol	−0.100	0.100	0.316	0.905	0.745	1.100
Smoking status	0.100	0.141	0.477	1.106	0.838	1.458

## Data Availability

The data used to support the findings of this study have not been made available because Prince Sattam Bin Abdulaziz University licensed our usage of the data for this study. Accordingly, we cannot disclose it to a third party. However, upon request, access to these data will be considered by the authors after the proper permission from Prince Sattam Bin Abdulaziz University.
